# Does Helium Pneumoperitoneum Reduce the Hyperinflammatory Response in Septic Animals during Laparoscopy?

**DOI:** 10.1155/2020/5738236

**Published:** 2020-03-12

**Authors:** Paulo Roberto Rodrigues Bicalho, Fernanda Magna Ribeiro, Pedro Henrique Ferreira Marçal, Daniel Gomes de Alvarenga, Fernando de Sá Silva

**Affiliations:** ^1^Departamento de Medicina, Universidade Federal de Juiz de Fora (UFJF), Campus Governador Valadares, Governador Valadares, MG, Brazil; ^2^Faculdade de Ciências da Saude, Universidade Vale do Rio Doce (Univale), Governador Valadares, MG, Brazil; ^3^Departamento Básico, Universidade Federal de Juiz de Fora (UFJF), Campus Governador Valadares, Governador Valadares, MG, Brazil

## Abstract

*Background/Aim*. An exacerbated reaction to peritoneal infection and attendant surgical procedures is characterized by an intense hyperinflammatory state, the magnitude of which is proportional to the severity of tissue injury. Laparoscopy generates lower levels of tissue damage compared with open surgery and should induce less pronounced immune responses. The aim of this study was to determine whether laparoscopy assisted by helium rather than carbon dioxide pneumoperitoneum would induce an attenuated inflammatory state in septic animals. *Materials and Methods*. Thirty-two Wistar rats were divided randomly into four equal groups, two of which were submitted to carbon dioxide or helium pneumoperitoneum-assisted laparoscopic cecal ligation and puncture (CLP) induced sepsis and subsequent abdominal lavage. Two control groups were submitted to identical laparoscopic procedures with carbon dioxide or helium as insufflator gas but without CLP. After 24 hours, serum levels of tumor necrosis factor alpha (TNF-*α*), interleukins 1 and 6 (IL-1 and IL-6, respectively), and cortisol were determined.

**Results:**

Mean concentrations of I L-1 and IL-6 in the groups of septic animals submitted to laparoscopy with carbon dioxide or helium pneumoperitoneum were not significantly different but were significantly higher than those of their respective non-CLP controls. In contrast, the levels of TNF-*α*), interleukins 1 and 6 (IL-1 and IL-6, respectively), and cortisol were determined.

**Conclusions:**

Laparoscopy with helium insufflation was similar to carbon dioxide in relation to the inflammatory response since levels of the proinflammatory TNF-*α*, IL-1, and IL-6 and of the anti-inflammatory cortisol were comparable for both gases.*α*), interleukins 1 and 6 (IL-1 and IL-6, respectively), and cortisol were determined.

## 1. Introduction

Peritonitis is the ultimate expression of a heterogeneous group of disorders of various degrees of severity that commonly occur in surgical practice. Diffuse fecal peritonitis is the most severe form of infection and leads rapidly to abdominal sepsis, which if left untreated, develops into septic shock, multiple organ failure, and death [1].

The inflammatory response induced by peritoneal infection produces physiological and immunological alterations in the patient resulting from complex interactions between the neuroendocrine, metabolic, and immune systems [2, 3]. Moreover, tissue trauma resulting from surgical incision, tissue dissection, manipulation of viscera and blood vessels, and exposure of the vascular endothelium is closely associated with systemic inflammatory response [4]. An exacerbated reaction to infection and any attendant treatment is characterized by an intense proinflammatory phase, and this hyperinflammatory state is detrimental to the individual because, paradoxically, it results in an immunosuppressive effect [5], the magnitude of which is proportional to the severity of tissue injury [6]. The resulting immunological impairment gives rise to various postoperative clinical complications that may be fatal [7].

Laparoscopic procedures generate lower levels of tissue injury compared with open surgery and should, theoretically, induce immune responses that are less pronounced [7]. On this basis, laparoscopy has been used increasingly in complex operations involving patients with abdominal sepsis [8]. Such procedures require the creation of pneumoperitoneum through insufflation of the abdominal cavity with a gas such as air, carbon dioxide, nitrous oxide, or helium [9].

Currently, carbon dioxide pneumoperitoneum is most commonly employed in both elective and emergency surgeries involving patients without comorbidities or sepsis. A number of researchers have reported that carbon dioxide pneumoperitoneum reduces the mortality in experimental animals with induced bacterial peritonitis [10–12], suggesting a protective effect of carbon dioxide against exacerbated inflammatory reactions. On the other hand, Chekan et al. [13] observed that carbon dioxide pneumoperitoneum reduced the intraperitoneal immune reaction in mice infected with *Listeria monocytogenes* in comparison with helium insufflation, thereby increasing the difficulty in controlling the spread of the infectious agent. It is known that the presence of carbon dioxide reduces the production of proinflammatory cytokines such as tumor necrosis factor alpha (TNF-*α*) and interleukins 1 and 6 (IL-1 and IL-6, respectively), suggesting that the gas temporarily inhibits macrophage activity via a mechanism that could be associated with the reduction of the local or systemic pH [14–18]. According to Hanly et al. [19], attenuation of the inflammatory response mediated by carbon dioxide pneumoperitoneum during laparoscopic surgery occurs via a mechanism involving peritoneal cell acidification.

Nevertheless, the advantages of standard-pressure carbon dioxide pneumoperitoneum over abdominal wall lift (with or without low-pressure pneumoperitoneum) or application of alternative insufflation gases have not yet been clearly established, especially with respect to local and systemic immune reactions [7]. In cases where inhibition of the inflammatory response by carbon dioxide has proven harmful to the immunological functions of the individual [13], an inert gas such as helium could be used as a substitute insufflator [20]. Some researchers [10, 18, 21] have stated that carbon dioxide is advantageous compared with helium in the creation of pneumoperitoneum in minimally invasive procedures since it is more efficient in limiting the inflammatory response and preserving the immune status of the individual. However, Sietses et al. [21] demonstrated that the levels of proinflammatory cytokines, such as IL-6, were similar in patients submitted to elective cholecystectomy involving carbon dioxide or helium pneumoperitoneum. Furthermore, Jacobi et al. [15] showed that in a rat model of peritonitis, insufflations with helium rather than carbon dioxide gave rise to a lower incidence of bacteremia and reduced systemic inflammation.

Considering that the pathophysiology and treatment outcomes of abdominal sepsis remains unclear, we hypothesized that helium pneumoperitoneum reduces the hyperinflammatory response in laparoscopy-treated rats with abdominal induced sepsis. The objectives of this study were to compare the systemic immune responses in rats with induced abdominal sepsis and treated by laparoscopy with either carbon dioxide or helium as insufflators and to determine the levels of the biomarkers of sepsis, namely cortisol, IL-1, IL-6, and TNF-*α*, in groups of rats submitted to different treatments. Our results enhance our understanding of the effects of different gases used in the creation of pneumoperitoneum.

## 2. Materials and Methods

The study was approved by the Animal Ethics Committee of the Universidade Federal de Minas Gerais (09th December 2015; protocol no. 417/2015) and conducted according to guidelines of humanitarian use of animals issued by National Council of Control of Animal Experimentation—CONCEA.

### 2.1. Animals and Experimental Design

The experiment was of randomized design and involved thirty-two12-week-old male Wistar rats supplied by the Animal House of the Universidade Federal de Minas Gerais. Animals were maintained in standard wooden cages under a 12 h photoperiod regime and supplied *ad libitum* with commercial rodent chow and water. Animals were distributed randomly into four experimental groups of eight rats each. Two groups were submitted to cecal ligation and puncture (CLP) induced sepsis and subsequent abdominal irrigation involving laparoscopic procedures with either carbon dioxide or helium pneumoperitoneum (groups SLC and SLH, respectively). Two control groups were submitted to identical laparoscopic procedures with either carbon dioxide or helium pneumoperitoneum (groups CLC and CLH, respectively) but without CLP. The mean weights (g ± standard errors) of the animals were 303.50 ± 29.97, 233.88 ± 63.33, 249.13 ± 21.72, and 304.63 ± 32.31 g for SLC, SLH, CLC, and CLH groups, respectively.

### 2.2. Surgical Procedures

**Figure 1:**
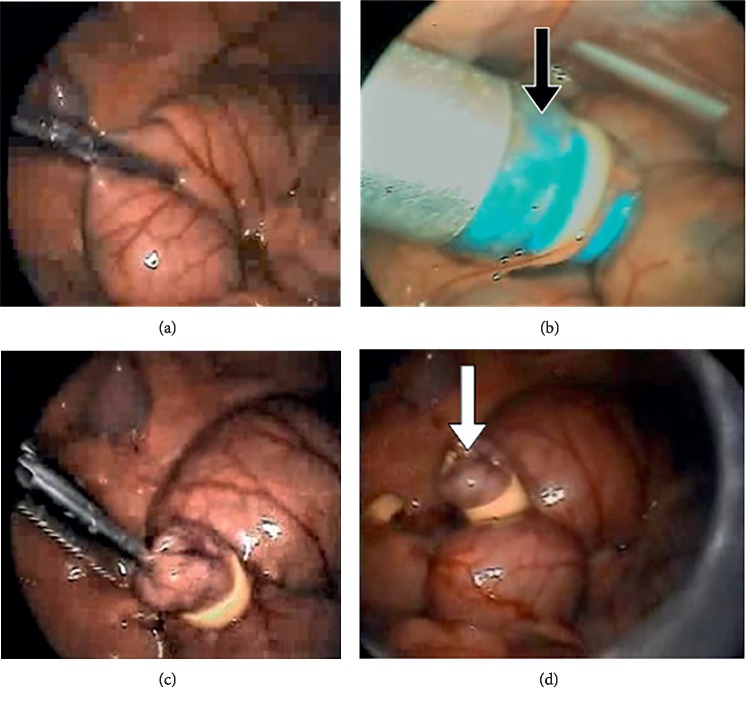
Laparoscopic cecal ligation and puncture showing (a) exposure of cecum; (b) clamping and traction of cecum into the elastic ligature (black arrow); (c) 30% sectioning of the cecal pouch; and (d) releasing of the fecal material into the abdominal cavity (white arrow).

Prior to surgery, all animals received a single intramuscular dose (30 mg/kg) of the antibiotic ceftriaxone (Rocephin; Roche, Rio de Janeiro, RJ, Brazil), and were subsequently anesthetized by intramuscular injection of a mixture of ketamine (40 mg/kg; Cristália Produtos Químicos e Farmacêuticos, Itapira, SP, Brazil) and xylazine (8 mg/kg; Schering-Plough, Cotia, SP, Brazil). Animals in groups SLC and SLH were submitted to laparoscopy with 15 min insufflation with either carbon dioxide or helium, as appropriate, in order to expose the cecum and allow CLP to be performed according to standard techniques (Figure 1) [22]. Animals in groups CLC and CLH were submitted to identical procedures except that no CLP was performed. Two hours after the start of surgery, animals of all groups underwent laparoscopic examination of the abdominal cavity involving a15 min insufflation with either carbon dioxide or helium, as appropriate. During this procedure, animals received two abdominal irrigations with 5 mL of 0.9% saline solution previously warmed to 37°C and the excess liquid was aspirated. A single dose of ibuprofen (10 mg/kg) was administered postoperatively to all experimental animals. At 24 h after surgery, a 2 mL sample of peripheral blood was collected from each animal by puncture of the inferior vena cava and transferred to a vial without anticoagulant. Blood samples were centrifuged twice at 2500 rpm for 10 min, following which serum was separated and stored in the freezer at −80°C for subsequent evaluation of the levels of proinflammatory biomarkers and cortisol. All experimental animals were then euthanized with an overdose of ketamine (120 mg/kg).

### 2.3. Immunoassays

Cortisol was assayed using a fluorescence polarization immunoassay (FPIA) kit, IL-1 was determined using a Quantikine™ Rat IL-1*β* kit (R&D Systems, Thermo Fisher Scientific, Minneapolis, MN, USA), and IL-6 and TNF-*α* were assessed using LEGEND MAX™ Rat IL-6 and TNF-*α* ELISA kits (BioLegend, San Diego, CA, USA). Serum samples were thawed at room temperature and aliquots (50 *μ*L) transferred to 96-well microplates together with 50 *μ*L of phosphate buffer and 18 *μ*L of the appropriate reagents in accordance with the instructions of the assay kit suppliers. Plates were incubated for 2 h at room temperature, washed with 500 *μ*L of buffer solution and centrifuged at 2500 rpm for 10 min. The supernatant was removed from individual wells by aspiration and diluted with 200 *μ*L of buffer, following which the difference in absorbance at 450 nm and 550 nm was determined using an ASYS Expert Plus spectrophotometer (Biochrom, Cambridge, UK).

### 2.4. Statistical Analysis

Statistical analyses were performed using IBM® SPSS® Statistics 19 software 1989. Data were submitted to the Kolmogorov–Smirnov test for normality and variables with normal distributions were compared using one-way analysis of variance (ANOVA) followed by Bonferroni's multiple comparison test or pairwise Student's *t*-test. Variables that presented nonnormal distributions were compared using either the multivariate Kruskal–Wallis test or the Mann–Whitney *U* test for comparison of two sample means. Differences between groups were considered statistically significant at *p* < 0.05.

## 3. Results

Assays conducted 24 h after the performance of CLP and laparoscopic examination revealed that the mean levels of anti-inflammatory cortisol and the proinflammatory cytokine TNF-*α* in SLC and SLH animals were not significantly different (*p* > 0.05) from those of their respective controls CLC and CLH (Table 1). Furthermore, the levels of cortisol in animals of the SLC and SLH groups were similar, as were those of TNF-*α*.

On the other hand, the mean concentrations of IL-1 in SLC and SLH animals were significantly higher (*p*=0.001 and 0.0001, respectively) than those of their respective controls, although the difference between animals of the SLC and SLH groups was not significant (*p*=0.1). In a similar manner, the mean IL-6 concentrations of SLC and SLH animals were significantly higher (*p*=0.001 and 0.009, respectively) than their respective controls although there was no significant difference (*p*=1) between the two groups with regard to the levels of this cytokine.

## 4. Discussion

In the present study, we assessed the levels of TNF-*α*, IL-1, IL-6 and cortisol in a murine model of abdominal sepsis and showed that the levels of the proinflammatory interleukins were significantly higher at 24 h after laparoscopic surgery in septic animals compared with their respective non-CLP controls. The elevation of these cytokines is compatible with the development of acute conditions and occurred regardless of the type of insufflator used in the laparoscopic procedure. Interestingly, no significant alterations were observed with respect to the levels of TNF-*α* and cortisol in the CLP groups compared with their respective non-CLP controls.

Acute peritonitis is associated with high rates of morbidity and mortality, and careful control of the source of infection remains key to a successful treatment [8, 23]. The use of minimally invasive surgical techniques, in particular percutaneous fluid drainage and laparoscopy, has been exceptionally valuable in cases of complex abdominal infections in patients with borderline physiological conditions such as diabetes, obesity, advanced age, and immunosuppression, where the negative impact of a laparotomy, although fundamental for the control of infection, may outweigh the benefit [23]. Although percutaneous drainage is feasible in most cases, some complex situations, such as unapproachable anatomical site or abscesses between loops, fistulas, and ischemic areas, require resection and catheter drainage is not practical [23]. For these reasons, laparoscopic procedures have gained popularity among surgeons for the performance of difficult operations on patients with abdominal sepsis [8].

Knowledge of the reaction of the human body, particularly the immune response, to minimally invasive procedures is important since it provides physicians with the foundations for decision-making regarding the risks and benefits of using novel approaches or access routes to treat their patients. Bench-to-bedside translational medicine in the area of abdominal sepsis is presently of considerable interest and the need to investigate specific aspects and intervening factors in the evolution of this syndrome has encouraged the search for animal models that reproduce the behavior of the human immune system. Several methods have been employed to induce peritonitis in animal models including intraperitoneal deposition of feces, inoculation of specific strains and amounts of microorganisms, and opening the gut and establishing a path of continuity to provide an endogenous source of fecal contamination [24]. The latter model includes the CLP technique, which was employed in the present study because it is considered the standard method for evaluating the immune response in sepsis situations and its correlation with invasive procedures [25]. The choice of experimental model is important for the clinical application of the study, since it is influenced by the variable of interest and affects the overall results obtained.

The severity of the clinical manifestations of sepsis depends not only on the virulence of the pathogens involved but also on the extent of the immune dysfunction developed by the host during progress of the disorder [5, 25]. Cytokines, especially TNF-*α* and IL-1, are important regulators of the immune response and their pathophysiological roles in sepsis have been widely studied [26]. The release of TNF-*α* from macrophages commences within 30 min after the inciting event, while IL-1 is released in a similar timeframe primarily from activated macrophages. Although these two cytokines act on different target cells (macrophages, endothelial cells, and neutrophils), both amplify inflammatory cascades by activating immune cells to secrete an array of immunoregulatory mediators such as proinflammatory cytokines, lipid mediators of inflammation, and reactive oxygen and nitrogen species. In this manner, TNF-*α* and IL-1 act synergistically to induce a shock-like state characterized by vascular permeability, severe pulmonary edema, hemorrhage, and fever.

The proinflammatory cytokine IL-6 is produced by a variety of cells, including macrophages, dendritic cells, lymphocytes, endothelial cells, fibroblasts, and smooth muscle cells, in response to stimulation by lipopolysaccharides, IL-1, and TNF-*α*. Concentrations of IL-6 are elevated in conditions such as surgical stress, trauma, multiple organ failure, and septic shock and peak after TNF-*α* and IL-1. Indeed, the plasma level of IL-6 is considered a reliable indicator of disease severity. However, despite its proinflammatory properties, IL-6 also has been shown to promote anti-inflammatory responses by inhibiting the release of TNF-*α* and IL-1 and enhancing the levels of anti-inflammatory mediators including IL-10, transforming growth factor beta (TGF-*β*) and the glucocorticoid hormone cortisol [26].

The concentration of cortisol is controlled through a cascade of events involving activation of the hypothalamic-pituitary-adrenal (HPA) axis, commencing with the release of corticotropin-releasing hormone (CRH) by the hypothalamus. This hormone promotes the production of adrenocorticotropic hormone (ACTH) in the pituitary gland, which in turn stimulates the synthesis of cortisol. In the acute phase of sepsis, the systemic concentration of cortisol is upregulated, and it has been shown that the level of cortisol within the first 24 h of the septic process is a good prognostic marker for mortality [27]. The mechanism of this increase in systemic cortisol levels is not totally clear but is believed to be induced either directly by augmentation of CRH and ACTH or indirectly by inhibition of the negative feedback action of cortisol on the HPA axis. In addition, some studies have demonstrated stimulatory effects of TNF-*α*, IL-1, and IL-6 on the HPA axis [28, 29].

Recent research on the pathophysiological mechanisms of sepsis has shown that the exacerbated release of proinflammatory cytokines (the so-called “cytokine storm”), such as TNF-*α*, IL-1, and IL-6, is counteracted by certain anti-inflammatory cytokines, including IL-10, transforming growth factor (TGF)-*β* and IL-4, in an attempt to restore immunological stability [26]. Deng et al. [5] used a mouse model involving CLP followed by intravenous challenge with *Pseudomonas aeruginosa* to demonstrate that the levels of cytokines increased considerably after surgery with a peak at 12 h, but after 24 h the levels of proinflammatory and anti-inflammatory cytokines diminished while the bacterial load remained high. These authors concluded that day one after CLP surgery was the turning point of overinflammation to immunosuppression.

Although carbon dioxide is the gas most commonly used in laparoscopic procedures [9], its advantages as an insufflator have been challenged. It is claimed that carbon dioxide insufflations contributes to the attenuation of the immune response through a mechanism involving acidification of the peritoneal cavity and the reversible inhibition of inflammatory cytokines IL-1 and TNF-*α* [14, 18]. In this context, Hanly et al. [10] reported that carbon dioxide pneumoperitoneum increased plasma IL-10 by 35% and reduced TNF-*α* by threefold compared with helium pneumoperitoneum, suggesting that mortality was reduce by IL-10-mediated downregulation of TNF-*α*. Other studies have shown that carbon dioxide insufflation increases survival and prevents mortality in murine models with induced peritonitis [10, 11] and could even display a protective effect [12]. However, some researchers have demonstrated that helium insufflation is better than carbon dioxide in preserving the immune response [13] and lowering bacteremia and local and systemic inflammation [15]. Other variables that influence local and systemic inflammation are the gas pressure used and the duration of laparoscopy. Schietroma et al. [30] showed levels of inflammatory markers (neutrophil elastase, IL-6 and IL-1, and C-reactive protein) significantly lower in laparoscopy adrenalectomy with reduced pressure CO_2_ pneumoperitoneum compared to the standard-pressure procedure, in a 24 h postoperative evaluation.

The results obtained in the present study showed that the inflammatory responses to abdominal sepsis in the murine model were similar regardless of whether carbon dioxide or helium was used in the creation of pneumoperitoneum since levels of the proinflammatory TNF-*α*, IL-1, and IL-6 and of the anti-inflammatory cortisol were comparable for both gases. The choice of the most appropriate gas for pneumoperitoneum remains in the hands of the physician since it depends on various factors including type of anesthesia, physiological compatibility, toxicity, ease of use, safety, delivery method, and cost.

Our study was limited to some extent by the small sample size employed and the short period (24 h) over which the biomarkers were assessed. In particular, the unexpected finding that TNF-*α* and cortisol levels had not risen within 24 h can be attributed to the limits of detection of the assay methods employed such that subtle alterations remained undetectable. The strength of our work resides in the appropriate controls employed. Further studies should address these limitations and focus on the investigation of interactions between helium and carbon dioxide pneumoperitoneum with respect to other variables.

## Figures and Tables

**Table 1:** Serum levels of biomarkers of sepsis in rats after 24 h of laparoscopic cecal ligation and puncture (CLP) induced sepsis assisted by either carbon dioxide or helium pneumoperitoneum.

Biomarkers		Laparoscopy with carbon dioxide insufflation			Laparoscopy with helium insufflation		
		SLC	CLC	*p* value^*∗*^	SLH	CLH	*p* value^*∗*^
Cortisol (ng/mL)							
Mean ± SD		0.72 ± 0.33	0.88 ± 0.28	>0.05	0.69 ± 0.27	0.56 ± 0.26	>0.05
Median		0.7	0.8		0.6	0.6	
Minimum		0.31	0.58		0.42	0.16	
Maximum		1.19	1.40		1.24	0.99	
Interleukin-1 (pg/mL)						
Mean ± SD		176.35 ± 73.07	48.45 ± 27.18	0.001	207.29 ± 81.65	41.99 ± 27.21	0.0001
Median		183.32	47.17		211.31	33.24	
Minimum		63.25	11.26		99.70	6.97	
Maximum		290.53	82.55		326.98	91.12	
Interleukin-6 (pg/mL)						
Mean ± SD		189.19 ± 69.10	41.50 ± 20.02	0.001	179.32 ± 111.64	56.11 ± 44.49	0.009
Median		175.05	33.75		166.51	40.14	
Minimum		72.15	17.50		41.59	14.09	
Maximum		305.09	74.11		361.63	146.44	
Tumor nuclear factor-*α* (ng/mL)						
Mean ± SD		16.59 ± 19.07	52.87 ± 62.85	>0.05	61.88 ± 47.52	39.91 ± 35.81	>0.05
Median		12.35	30.52		92.03	36.01	
Minimum		0	0		0	0	
Maximum		48	168		103	83	

^*∗*^Differences considered statistically significant at *p* < 0.05 according to Kruskal–Wallis or Mann–Whitney *U* tests. SD, standard deviation; SLC, sepsis laparoscopy carbon dioxide pneumoperitoneum; CLC, control laparoscopy carbon dioxide pneumoperitoneum; SLH, sepsis laparoscopy helium pneumoperitoneum; and CLH, control laparoscopy helium pneumoperitoneum.

## Data Availability

The data used to support the findings of this study are included within the article (Table 1).
